# Progestin-primed ovarian stimulation vs mild stimulation in women with advanced age above 40: a retrospective cohort study

**DOI:** 10.1186/s12958-019-0518-3

**Published:** 2019-11-09

**Authors:** Qian Peng, Xiang Cao, Jing Wang, Lin Wang, Jun Xu, Xiaowei Ji, Suying Liu, Jin Zhu, Xi Dong

**Affiliations:** 1 Reproductive medicine centre, Zhongshan hospital, Fudan University, Shanghai, China; 2 Department of Obstetrics and Gynecology, Shanghai Obstetrics and Gynecology Hospital, Fudan University, Shanghai, China

**Keywords:** Progestin-primed ovarian stimulation, Mild stimulation, Advanced maternal age, Embryo quality

## Abstract

**Background:**

Previous studies have demonstrated that progestin-primed ovarian stimulation (PPOS) protocol was a feasible and efficient method in in vitro fertilization (IVF) cycle. However, its application in women with advanced age has not been determined yet. The purpose of this study was to investigate its efficacy in women aged ≥40 years old.

**Methods:**

This retrospective cohort study included patients with ages of ≥40 years old at the time of ovarian stimulation. The embryonic and clinical outcome of mild stimulation and PPOS were compared. Primary outcome was top-quality embryo rate on day 3, and secondary outcome was clinical pregnancy rate.

**Results:**

Baseline characteristics of patients was similar in mild stimulation (122 cycles) and PPOS (47 cycles). No significant difference was found in the number of retrieved and mature oocytes and the fertilization and cleavage rates. Of interest, the rate of top-quality embryos was significantly higher in PPOS group (50.08% vs 33.29%, *p* = 0.015), with an increasing trend of viable embryo rate (73.55% vs 61.16%). A greater amount of gonadotropin was observed in PPOS group (2061.17 ± 1254.63 IU vs 1518.14 ± 547.25 IU, *p* < 0.05) in spite of comparable duration of stimulation. After FET cycle, no significant difference was found in the clinical pregnancy rates between mild stimulation (12.5%) and PPOS group (16.7%).

**Conclusions:**

Higher percentage of top-quality embryos on Day 3 and comparable clinical pregnancy rate was obtained in PPOS protocol, which could be considered as a feasible ovarian stimulation protocol in women aged above 40 years old.

## Background

Since universal two-child policy took effect in 2015 in China, the number of women with advanced age over 40 undergoing IVF is rapidly growing. It is well known that oocyte yield and quality decrease with increased maternal age. Oocyte quality is the predominant determining factor for embryo quality, thus it affects the clinical pregnancy and live birth rates. According to the report from Society for Assisted Reproductive Technology (SART) in 2015 in the USA, live birth rate per egg retrieval cycle was only 11.1 and 3.6% for women aged 41–42 and > 42 group, respectively. Cumulative live birth rate was slightly higher for 41–42 group (12.6%) and > 42 group (3.9%) (https://www.sartcorsonline.com). Increased live birth rate and cumulative live birth rate were associated with increased number of retrieved oocytes [1, 2], which was affected by different ovarian stimulation protocols in IVF cycles. In the SART report system, there were four categories with different protocols including minimal stimulation, natural cycle, conventional stimulation and in vitro maturation. Unlike conventional stimulation protocol using high doses of gonadotropins, lower doses of gonadotropins were used in mild stimulation protocol based on the concept that more competent oocytes can be developed. Although mild stimulation protocol suffered from fewer numbers of oocytes or embryos obtained, it was proven to be safe and cost effectiveness. In addition, the cumulative pregnancy outcome was comparable to those that used conventional stimulation in normal and poor ovarian responders [3, 4].

PPOS protocol was initially employed by Prof. Kuang in 2015 [5]. The protocol has been demonstrated to result in good embryos and clinical outcomes in normal [5, 6] and poor ovarian responders [7, 8], and polycystic ovary syndrome (PCOS) patients [9]. The purposes of progesterone introduction into ovarian stimulation are to block luteinizing hormone (LH) surge. The LH surge is caused by the rising plasma estradiol and triggers ovulation, and a premature LH surge could lead to down-regulation of oocyte yield. Medroxyprogesterone acetate (MPA) [5, 10], utrogestan [6] and dydrogesterone (DYG) [11] could be successfully served as an adjuvant to human menopausal gonadotropin (HMG) and effectively prevent premature LH surge. Gonadotropin-releasing hormone (GnRH) antagonist could also be used, but about 38.3% of the patients suffered from premature luteinization [12]. In poor responders, the efficacy of PPOS protocol was only compared to natural cycle [8] and GnRH antagonist protocol [7], and progestin priming was proved to have no adverse or even better effect on clinical outcome. According to the Bologna criteria [13], women with advanced age (≥40) is the most relevant risk factor for poor ovarian response. Therefore, the aim of the current retrospective cohort study was to compare the efficacy of PPOS and mild stimulation protocols in women with advanced age.

## Methods

### Database

This retrospective cohort study was conducted in the reproductive medicine centre of Zhongshan Hospital in Shanghai. Women with ages ≥40 years undergoing IVF/ Intracytoplasmic sperm injection (ICSI) and subsequent frozen-thawed embryo transfer (FET) cycles between April 2016 and January 2019 were recruited. Written consents were obtained from all participants after counseling and IVF treatments. The patients were divided into two groups: mild stimulation group using clomiphene citrate (CC) with HMG and PPOS group. A proportion of them was followed by FET cycle.

### Ovarian stimulation protocol, embryo culture and vitrification

The mild stimulation protocol was performed as followed: starting from Day 3 of menstrual cycle, patients received oral CC (Codal Synto Ltd., Cyprus) at 100 mg/d and HMG injection (Lizhu Pharmaceutical Trading Co., Zhuhai, China) at 150–225 IU/d daily. The patients in PPOS protocol received oral DYG (Duphaston; Abbott Biologicals B.V., Netherlands) at 20 mg/d and HMG injection at 150–225 IU/d daily from menstrual cycle Day 3 onwards. In both methods, the dosage of HMG was adjusted according to follicular diameter and estradiol level. The final oocyte maturation was triggered by intramuscular injection of human chorionic gonadotropin (hCG; Lizhu Pharmaceutical Trading Co.) at 5000–10000 IU or subcutaneously injection of triptorelin acetate (200 μg) after evaluation of estradiol and progesterone levels in combination with the number of follicles with diameter ≥ 16 mm.

Oocyte retrieval was performed under the guidance of transvaginal ultrasound. Follicles with > 10 mm in diameter were aspired at 34–36 h after ovulation trigger. Oocytes were fertilized by IVF/ICSI in G-IVF™ Fertilization media (Vitrolife, Göteborg, Sweden) with 10% human serum albumin (HSA, Vitrolife). Embryos were cultured in either one step continuous single culture media (CSC; Irvine Scientific, CA, USA) or sequential culture media G-1™ / G-2™ medium (Vitrolife) with 10% HSA.

Embryo morphology was assessed and graded on Day 3 according to Cutting’s criteria [14]. The embryos recorded as higher than 5c (2/2) were considered as viable embryo, while those recorded as higher than 6c (3/4) were considered as top-quality embryo. Generally, two top-quality embryos were vitrified on Day 3, and supernumerary embryos were cultured continuously until blastocyst stage on Day 5 before vitrification. The vitrification procedure was performed following standard protocols using Irvine Scientific Freeze Kit (Irvine Scientific) with home-made straw carrier system.

### FET cycle, endometrial preparation and luteal support

In some patients, hormonal treatment was used to prepare the endometrium for the FET cycles. Starting from menstrual cycle Day 3, patients received oral estradiol valerate (Progynova; Bayer, Germany) daily. From Day 10 onwards, serum hormone levels were measured, and endometrium growth were monitored by transvaginal ultrasound. When endometrial thickness ≥ 7 mm and progesterone < 3.18 nmol/L were observed around Day 14, oral DYG (20 mg/d) and vaginal progesterone (600 mg/d) were applied. Embryo transfer was scheduled on Day 3, 4 and 5. Luteal support was maintained until 8–10 weeks of gestation or negative β-hCG detection 2 weeks after transfer. Clinical pregnancy was defined as the presence of gestational sac determined by ultrasound around 7 weeks. FET cycles that included mixed embryos from different protocols were removed from this study.

### Data analysis

The primary outcome was top-quality embryo rate on Day 3. The secondary outcome was clinical pregnancy rate. Statistical analysis was performed using SPSS (Version 22, SPSS Inc., Chicago, IL, USA). Continuous variables were presented as mean values and standard deviations and were compared using one-way ANOVA or Mann–Whitney test if applicable. Chi-square test and Fisher’s exact test were used to compare percentages of qualitative variables and clinical outcomes. A logistic regression analysis was performed to assess for the associations between variables and pregnancy outcome. The data was shown as odds ratios (ORs) and 95% confidence intervals (CIs).

## Results

A total of 169 cycles including mild stimulation group (122 cycles) and PPOS group (47 cycles) were selected from our database during the study time period and were retrospectively examined. By the end of the research period (January 31, 2019), 74 FET cycles were completed, in which 56 cycles was from mild stimulation group and 18 cycles was from PPOS group. Demographic and basal characteristics of these patients are presented in Table 1. No difference in age, body mass index (BMI), basal follicle stimulating hormone (FSH) and LH levels, factors and durations of infertility and percentages of primary infertility was found between the two groups. However, antral follicle count (AFC) was a bit lower (7.09 ± 3.29 vs 8.23 ± 3.17, *p* = 0.039) while the number of previous IVF attempts was higher (2.79 ± 1.94 vs 2.00 ± 2.11, *p* = 0.028) in PPOS group when compared to mild stimulation group. Besides, basal estradiol (135.06 ± 84.75 vs 192.91 ± 149.42, *p* = 0.006) and progesterone (1.25 ± 0.85 vs 0.56 ± 0.30, *p* < 0.001) levels were significantly different between mild stimulation and PPOS groups.

**Table 1:** Demographics and basal characteristics of patients in this study

Characteristics	Mild stimulation	PPOS	*p* value
Age	43.32 ± 2.49	43.15 ± 2.56	0.692
Basal FSH (mIU/ml)	8.70 ± 2.51	7.91 ± 2.21	0.061
Basal LH (mIU/ml)	4.58 ± 1.60	4.68 ± 1.98	0.742
Basal Estradiol (pmol/L)	135.06 ± 84.75	192.91 ± 149.42	0.006
Basal Progesterone (nmol/L)	1.25 ± 0.85	0.56 ± 0.30	< 0.001
BMI	22.63 ± 2.68	21.95 ± 2.63	0.137
AFC	8.23 ± 3.17	7.09 ± 3.29	0.039
Infertility factor (%)			0.500
Female	93(76.2%)	36(76.7%)	/
Male	15(12.3%)	3(6.4%)	/
Both	2(1.6%)	2(4.3%)	/
Unexplained	12(9.8%)	6(12.8%)	/
Primary infertility (%)	21(17.2%)	9(19.1%)	0.944
Infertility duration	5.78 ± 4.49	6.33 ± 5.95	0.810
Previous IVF attempts	2.00 ± 2.11	2.79 ± 1.94	0.028

Data was present as mean ± SD

The parameters and characteristics of the cycles in both groups were shown in Table 2. No obvious difference was observed in duration of ovarian stimulation, but the total amount of gonadotropin was significantly higher in PPOS group (2061.17 ± 1254.63, *p* < 0.001). Moreover, LH, estradiol and progesterone levels on the trigger day were significantly lower in PPOS group (5.14 ± 2.81, 5241.02 ± 2712.04 and 1.83 ± 2.57, respectively, *p* < 0.05). Meanwhile, the incidence of premature LH surge (defined as a LH level ≥ 10 IU/L and a progesterone level ≥ 3.18 nmol/L on trigger day [15]) was significantly lower in PPOS group. However, cancellation rate, number of retrieved and mature oocytes, fertilization and cleavage rates were comparable between mild stimulation and PPOS groups. The viable embryo rate was higher in PPOS group (73.55 ± 36.58% vs 61.16 ± 41.64%), but the difference was not significant. However, top-quality embryo rate was significantly higher in PPOS group when compared with mild stimulation group (50.08 ± 41.65% vs 33.29 ± 39.32%, *p* = 0.015). Linear regression analysis showed no significant association of top-quality embryo rate with progesterone level on the trigger day (*p* = 0.164).

**Table 2:** Characteristics of cycle parameters in both the group

Variables	Mild stimulation	PPOS	*p* value
Duration of ovarian stimulation (days)	9.06 ± 2.05	8.49 ± 2.03	0.108
Total dose of gonadotropin (IU)	1518.14 ± 547.25	2061.17 ± 1254.63	< 0.001
FSH on trigger day (mIU/ml)	16.76 ± 6.90	16.51 ± 4.80	0.432
LH on trigger day (mIU/ml)	9.38 ± 4.72	5.14 ± 2.81	< 0.001
Estradiol on trigger day (pmol/L)	6959.59 ± 4399.75	5241.02 ± 2712.04	0.013
Progesterone on trigger day (nmol/L)	2.61 ± 1.78	1.83 ± 2.57	0.028
Premature LH surge-no. (%)	15(12.3%)	1(2.1%)	0.044
Cancelation rate (%)	31(25.4%)	7(14.9%)	0.207
Number of oocytes retrieved	3.57 ± 2.77	3.72 ± 2.76	0.740
Number of mature oocytes	3.08 ± 2.39	2.87 ± 2.20	0.602
Fertilization rate (%)	79.05 ± 31.50	77.36 ± 30.74	0.754
Cleavage rate (%)	87.25 ± 30.25	90.06 ± 29.43	0.585
Viable embryo rate (%)	61.16 ± 41.64	73.55 ± 36.58	0.095
Top-quality embryos rate (%)	33.29 ± 39.32	50.08 ± 41.65	0.015

Data was present as mean ± SD

In the subsequent FET cycle, clinical pregnancy rates were comparable between mild stimulation (12.5%, 56 cycles) and PPOS (16.7%, 18 cycles) groups (Table 3). The number of transferred embryos at different stages and the endometrial thickness were not statistically different between mild stimulation and PPOS groups.

**Table 3:** Clinical outcomes of FET cycles

Variables	Mild stimulation	PPOS	*p* value
Number of thawed cycles	56	18	
Number of transferred embryos at different stage			0.134
Number of Cleavage-stage embryo transferred-no. (%)	34(60.7%)	11(61.1%)	/
Number of Blastocyst transferred-no. (%)	18(32.1%)	3(16.7%)	/
Number of Cleavage-stage and Blastocyst embryo transferred-no. (%)	4(7.1%)	4(22.2%)	/
Endometrial thickness (mm)	9.78 ± 1.93	9.00 ± 1.97	0.140
Clinical Pregnancy rate - no. (%)	7(12.5%)	3 (16.7%)	0.697

A binary logistic regression model was performed (Table 4). The dependent variable was clinical pregnancy outcome, and the independent variables included maternal age, BMI, duration of infertility, number of previous IVF attempts and transferred embryos per cycle, endometrial thickness and the ovarian stimulation type. Significant negative effect of maternal age (OR = 0.644, 95% CI: 0.418–0.993) was found. There was a trend of adverse effect of duration of infertility (OR = 0.848, 95% CI:0.709–1.015), however, the result was not significant (*p* = 0.073). Furthermore, no effect was identified in other variables.

**Table 4:** Logistic regression analysis of clinical pregnancy outcome in the study

Independent variables	OR [95% CI]	*p* value
Maternal age	0.644[0.418,0.993]	0.046
BMI	0.946[0.662,1.352]	0.761
Duration of infertility	0.848[0.709,1.015]	0.073
Number of previous IVF attempts	0.924[0.631,1.354]	0.685
Number of transferred embryos per cycle	0.975[0.238,3.996]	0.971
Endometrial thickness	1.289[0.851,1.954]	0.231
Mild stimulation vs PPOS	0.544[0.137,2.159]	0.386

## Discussion

The present study evaluated the efficacy of PPOS protocol in women with advanced age above 40, when compared to mild stimulation using CC + HMG. The data suggested that PPOS protocol achieved better embryonic outcome, as demonstrated by the increased number of top-quality embryos obtained on day 3. Although statistically differences were observed in basal estradiol and progesterone between the groups, their levels were within normal range. AFC and previous IVF attempts were even worse in PPOS group.

The estradiol level was found higher while the total dose of gonadotropin was found lower on the trigger day in mild stimulation group. This may be due to the fact that, clomiphene citrate binds to the estrogen receptors on the hypothalamus and alters the negative feedback effect of estrogen, therefore induces the secretion of GnRH [16], which resulted in the higher level of estradiol in the mild stimulation group. In PPOS group, dydrogesterone application leads to pituitary suppression, which inhibits GnRH secretion [11]. Therefore, higher dosage of gonadotropin was required during ovarian stimulation.

Meanwhile, the progesterone level on the trigger day was higher in mild stimulation group as well. It has been demonstrated that estradiol levels on the day of hCG administration were significantly increased in women with progesterone elevation within 0.8–1.1 ng/ml [17]. The progesterone concentration in the present study was 2.61 ± 1.78 nmol/L (0.82 ± 0.56 ng/ml) in mild stimulation group. The exact cause of progesterone elevation at the end of the follicular phase during ovarian stimulation of IVF is still obscure. It could be due to premature LH surges, which is caused by the actions of estradiol induced by gonadotrophins. Premature luteinization exists in up to 15–20% of IVF cycles, which could lead to cancellation of cycles [18]. Consistently, the percentage of premature LH surge was significantly higher in mild stimulation when compared with PPOS group. There was also a trend of higher cancelation rate, though it was not statistically significant. It is well-known that IVF pregnancy outcome decreased dramatically in women with advanced age. They suffered from higher risk of premature luteinization, which could be due to changes of gene expressions related to gonadotropin activity. Previous study reported the down-regulation of FSH receptor (*FSHR*) but up-regulated LH receptor (*LHCGR*) and progesterone receptor (*PGR*) gene expressions in granulosa cells in women aged more than 43 [19]. The increased expression of LHCGR and PGR and decreased FSHR has been reported in luteinized granulosa cells [20–22]. Progesterone has been reported to play an important role in oocyte nuclear and cytoplasmic maturation and developmental competence [23–25]. The better embryonic outcome with progestin administration in PPOS protocol may be due to modulated interaction between progesterone and its receptor in aged female. However, the impact of endogenous progesterone level on the trigger day on embryo quality is still controversial. A large systematic review and meta-analysis of over 60,000 cycles study has demonstrated that the detrimental effect of progesterone concentration on clinical pregnancy started from range of 0.8–1.1 ng/ml. However, such effect was observed only in women undergoing fresh IVF cycles but not FET cycles, because it was hypothesized that it was through its action on the endometrium [17]. In the current study, FET cycles were used and the progesterone level in the mild stimulation group did not exceed the marginal value reported in that study. Another report showed that premature progesterone elevation was not associated with embryo quality [26]. In agreement with that study, our result also showed no association between the progesterone level with top-quality rate. There was one report suggesting a negative association between these two parameters [27]. However, the cutoff value of progesterone detrimental to embryo quality was 2.0 ng/ml in the study, which was much higher than that in the mild stimulation group in our study. Taken together, lower LH level, blockage of premature LH surge and better embryonic outcome indicated that PPOS could be served as a feasible ovarian stimulation method for aged women.

The first randomized cohort study of PPOS was reported by Prof. Kuang in 2015 with the combination of MPA and HMG (150–225 IU/d) administration from menstruation day 3 onwards [5]. Although the duration of stimulation was significantly longer and total dose of HMG was significantly higher in PPOS when compared to conventional short protocol in normal responders, no difference was found in the number of mature oocytes and frozen embryos between the two groups [5]. There was also no difference found in the implantation rate, clinical pregnancy rate, miscarriage rate and live birth rate after FET cycles. However, a recent retrospective study has shown significantly higher matured oocytes rate, fertilization rate, good-quality embryo rate, clinical pregnancy and live birth rates in PPOS protocol when compared to GnRH antagonist protocol in poor responders [28]. The good-quality embryo rates in their PPOS group was 70% [28], which was higher than that of 50% in our study. This could be due to more stringent criteria employed during our embryo morphology assessment on day 3. The good-quality embryos in their study referred to those with at least six blastomeres and fragments < 50% [28], while ours were defined as embryos with at least six blastomeres and fragments < 20%. Meanwhile, same PPOS protocol was compared to natural cycle in poor responders with diminished ovarian reserve in a non-randomized prospective cohort study [8]. Their result showed that half of the patients in natural cycle group had LH surges while none was observed in PPOS group, and the number of mature oocytes and viable embryos was significantly higher in PPOS group. The proportion of cycles with at least one viable embryo in their PPOS group was 50.0%, while the viable embryo rate in our study was 73%. Unlike the protocols in our study, they used exogenous progesterone and low dose of HMG in the late follicular phase, and the result showed that progestin primed minimal stimulation protocol could also efficiently control the development of dominant follicle and embryo quality in poor responders [8]. In patients with high BMI, higher implantation rate, clinical pregnancy rate and live birth rate were associated with PPOS protocol when compared with conventional short protocol [29]. The efficacy of PPOS was also compared to short agonist protocol in a pilot study of 60 PCOS patients [9]. In contradiction to previous result in natural cycle [8], no difference in the number of oocytes collected and ongoing pregnancy rates but a higher total dose of HMG was found in the PPOS group of PCOS patients. Therefore, the controversy still exists due to different outcomes in different populations. DYG was used as exogeneous progestin in this study. Even though greater assumption of gonadotropin was observed in PPOS group, it is cost-effective as the price of HMG is comparatively low. DYG is an alternative to MPA in PPOS protocol in a recent prospective randomized controlled trial (RCT) [11]. The study showed that no significant difference was found between DYG and MPA in terms of number of oocytes retrieved, the viable embryo rate per oocyte and the clinical pregnancy rate.

The use of progesterone to block the LH surge has been summarized in a recent review [30]. Either endogenous with luteal phase stimulation and exogenous with PPOS was confirmed effective. Several studies have demonstrated that high progesterone in follicular and luteal phase stimulation had no adverse effect on oocyte and embryo qualities. Retrospective study demonstrated that endogenous progesterone alone was sufficient to block the LH surge and agonist or antagonist was unnecessary. Implantation rates were similar in luteal stimulation and follicular stimulation [31, 32]. Multiple stimulation protocols have been derived from ovarian stimulation protocol under high progesterone, such as ‘duostim’ [33] or ‘Shanghai protocol’ [34]. It has an advantage for fertility perseveration in the urgent context of oncology. However, the application and cost-effectiveness of the stimulation protocol for poor responders was still undetermined due to retrospective studies with few patients. Our study also suffered from small sample size and its nature of retrospective design. Future RCT study is required. Besides, the primary outcome was top-quality embryos on day 3, which may be not the best parameter to predict the prognosis of patients undergoing IVF cycle. The current study would provide an alternative approach for patients with advanced ages in obtaining embryos with better quality, which potentially alleviate their stress during IVF treatment cycles.

## Conclusions

In conclusion, our data showed that the use of progesterone during ovarian stimulation could efficiently block the LH surge in women with advanced age, and it did not affect the number of oocytes collected but resulted in better embryonic outcome on day 3. Such protocol had a promising application in aged patients who wished to reserve embryos in a short period and shorten their time to pregnancy.

## Data Availability

The datasets used and/or analyzed during the current study are available from the corresponding author on reasonable request.

## Abbreviations

AFC: Antral follicle counting
BMI: Body mass index
CC: Clomiphene citrate
CIs: Confidence intervals
DYG: Dydrogesterone
FET: Frozen-thawed embryo transfer
FSH: Follicle stimulating hormone
FSHR: FSH receptor
GnRH: Gonadotropin-releasing hormone
hCG: Human chorionic gonadotropin
HMG: Human menopausal gonadotropin
HSA: Human serum albumin
ICSI: Intracytoplasmic sperm injection
IVF: In vitro fertilization
LH: Luteinizing hormone
LHCGR: LH receptor
MPA: Medroxyprogesterone acetate
ORs: Odds ratios
PCOS: Polycystic ovary syndrome
PGR: Progesterone receptor
PPOS: Progestin-primed ovarian stimulation
SART: Society for Assisted Reproductive Technology
